# Machine learning-driven prediction of intratumoral tertiary lymphoid structures in hepatocellular carcinoma using contrast-enhanced CT imaging and integrated clinical data

**DOI:** 10.3389/fonc.2025.1652509

**Published:** 2025-11-10

**Authors:** Jun Wu, Zhifan Zuo, Lin Na, Wei Zhang, Yang Guo, Ziwei Zhu, Qiongyuan Ren, Weng Kung Peng, Lei Han

**Affiliations:** 1 Department of Hepatobiliary Surgery, The General Hospital of Northern Theater Command, Shenyang, Liaoning, China; 2 Gynecological Radiotherapy Ward, Liaoning Provincial Cancer Hospital, Shenyang, Liaoning, China; 3 Department of Central Laboratory, The First Affiliated Hospital of China Medical University, Shenyang, Liaoning, China; 4 Dalian Medical University, The General Hospital of Northern Theater Command Training Base for Graduate, Shenyang, Liaoning, China; 5 Center for Quantum Information and Quantum Biology, Institute of Advanced Co-Creation Studies, The University of Osaka, Osaka, Japan

**Keywords:** hepatocellular carcinoma, intratumoral tertiary lymphoid structures, machine learning, radiomics, contrast-enhanced CT

## Abstract

**Purpose:**

We developed a machine learning framework to predict the presence of tertiary lymphoid structures (TLSs) within tumors in patients with hepatocellular carcinoma (HCC). This framework uses computed tomography (CT) imaging and clinical data collected before surgery, providing a noninvasive method for prediction.

**Methods:**

We conducted a retrospective analysis of HCC patients who underwent surgery at the General Hospital of the Northern Theater Command’s Hepatobiliary Surgery Department between January 2017 and October 2024. Using Python software, we extracted radiomic features from preoperative CT images (arterial and portal venous phases). We then selected features associated with intratumoral TLSs using statistical methods, including intraclass correlation coefficient (ICC), Pearson correlation, t-tests, and LASSO regression. Three models were developed—clinical, radiomics, and combined—using machine learning techniques and independent clinical predictors. A predictive nomogram was created and evaluated using the area under the ROC curve (AUC) and calibration analysis.

**Results:**

Our study included 171 HCC patients, with 80 showing negative and 91 showing positive expression of intratumoral TLSs. Multivariate analysis identified the albumin-bilirubin (ALBI) score as an independent predictor of intratumoral TLSs expression. The combined model demonstrated the highest predictive accuracy, with AUCs of 0.947 in the training set and 0.909 in the validation set, outperforming both the clinical (AUC: 0.709 training, 0.714 validation) and radiomics (AUC: 0.935 training, 0.890 validation) models.

**Conclusion:**

Our combined machine learning model, which integrates preoperative CT imaging and clinical data, provides an accurate, noninvasive method for assessing intratumoral TLSs expression in HCC. This tool has the potential to enhance clinical decision-making, guide therapeutic planning, and facilitate personalized treatment strategies for HCC patients.

## Introduction

Hepatocellular carcinoma (HCC) constitutes the predominant subtype of liver cancer, accounting for 75–85% of cases (1). Globally, it ranks as the sixth most commonly diagnosed malignancy and the third leading cause of cancer-related mortality (2). Although radical liver resection represents the primary curative treatment for HCC (3), most patients are diagnosed at intermediate or advanced stages, rendering them ineligible for surgical intervention (4). The heterogeneous nature of HCC, combined with its resistance to conventional radiotherapy and chemotherapy, contributes to a poor overall prognosis (5, 6).

Recent progress in targeted therapies and immunotherapies has introduced novel therapeutic options for HCC management. Immune checkpoint inhibitors, particularly those targeting programmed death-1 (PD-1) and cytotoxic T lymphocyte-associated antigen-4 (CTLA-4), have emerged as critical components of contemporary HCC research (7, 8). In a phase III clinical trial, nivolumab monotherapy achieved an objective response rate of 18.3% in patients with advanced HCC, demonstrating clinical efficacy and a favorable safety profile (9). Despite its superior efficacy relative to other treatments, immunotherapy is limited by significant interpatient variability in response (10). Consequently, the development of predictive biomarkers for immunotherapy response remains essential for optimizing clinical decision-making in HCC.

Tertiary lymphoid structures (TLSs) have recently emerged as a promising focus in cancer immunotherapy research due to their potential to improve treatment outcomes (11). These organized aggregates of immune cells develop in non-lymphoid tissues, driven by chronic inflammatory conditions such as cancer, autoimmune disorders, or persistent infections (12). Structurally, TLSs recapitulate the architecture of lymph nodes, featuring a core of B cells (CD20+) surrounded by T cells (CD3+), which collectively facilitate the initiation and coordination of adaptive immune responses (13). TLSs can remodel the local immune landscape by promoting the infiltration of anti-tumor effector cells while simultaneously suppressing pro-tumorigenic populations (14). This evidence positions TLSs as localized hubs for priming anti-tumor immunity, underscoring their significance as a key area of investigation for advancing therapeutic strategies.

Emerging evidence highlights the pivotal role of TLSs as central hubs for initiating systemic antitumor immune responses. In melanoma, dense perivascular clusters of CD8+ T cells surrounding TLSs underscore their function as activation sites for tumor-directed immunity (15). Mature TLSs have been identified as a key biomarker for predicting immunotherapy efficacy (16), with soft tissue sarcomas harboring TLSs exhibiting superior responses to immune checkpoint blockade (17). Clinically, TLSs presence correlates with prolonged overall survival across diverse malignancies, including gastric, cervical, and breast cancers (18–22). In HCC, intratumoral TLSs are linked to reduced early recurrence post-resection and better prognosis in early-stage disease (23). Furthermore, the spatial distribution and density of TLSs in intrahepatic cholangiocarcinoma offer a refined immune-based stratification system for prognostic assessment (24). Given these findings, preoperative prediction of TLSs presence in HCC holds significant clinical value, enabling more accurate prognosis estimation, personalized therapeutic strategies, and optimized treatment selection.

Currently, TLSs can only be definitively identified through postoperative histopathological analysis. However, HCC is primarily diagnosed non-invasively using imaging techniques such as computed tomography (CT) or magnetic resonance imaging (MRI), which often eliminates the need for pathological confirmation. Given the prognostic and therapeutic significance of TLSs, there is an urgent need for a non-invasive, efficient preoperative tool to predict the presence of intratumoral TLSs in HCC.

Machine learning (ML) has emerged as a powerful tool for disease diagnosis and treatment optimization, combining high accuracy with computational efficiency (25). Radiomics—a field focused on extracting and analyzing quantitative imaging features to uncover hidden biological patterns—has gained significant traction in clinical research due to its potential to predict disease onset, progression, and outcomes (26, 27). In liver cancer, radiomics has already demonstrated promising results. For instance, Feng et al. developed an MRI-based radiomics model for the preoperative prediction of microvascular invasion (MVI), achieving an area under the curve (AUC) of 0.83 (28). Similarly, Li et al. constructed a multiparametric CT-derived radiomics nomogram to identify the massive macrotrabecular HCC subtype with high accuracy (29).

Building on these advances, this study focuses on intratumoral TLSs in HCC and aims to develop a preoperative prediction model using machine learning algorithms applied to contrast-enhanced CT imaging and clinical data. The model is designed to improve clinical decision-making, advance precision medicine, enable personalized preoperative risk stratification, and optimize therapeutic strategies for HCC patients.

## Materials and methods

**Figure 1:**
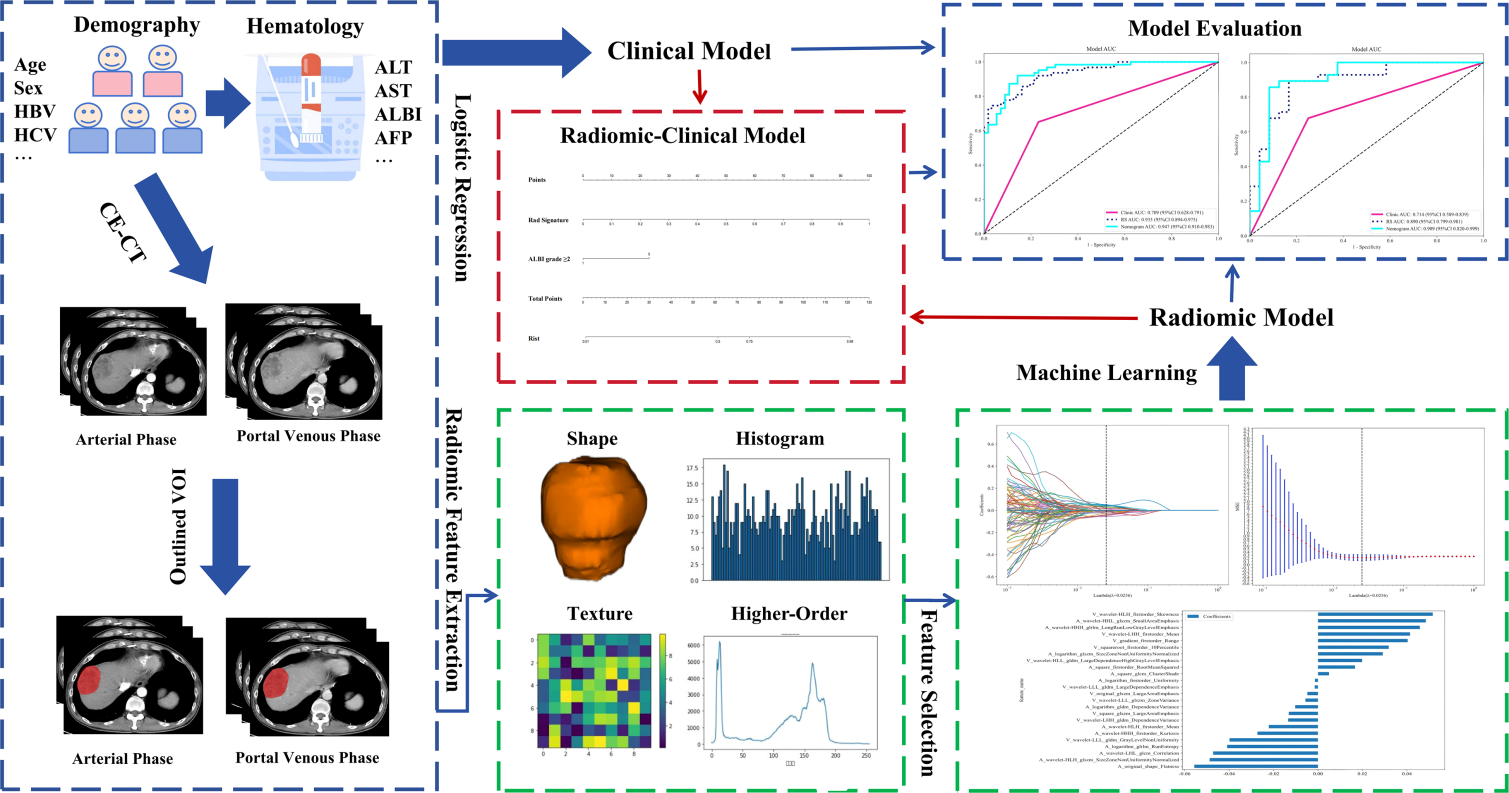
Workflow for development of the clinical-radiomics model. HBV, hepatitis B virus; HCV, hepatitis C virus; ALT, alanine aminotransferase; AST, aspartate aminotransferase; AFP, alpha-fetoprotein; CE-CT, contrast-enhanced computed tomography; VOI, volume of interest; ALBI, albumin-bilirubin; RS, Rad Signature.

Workflow for the development of the clinical-radiomics model is shown in **Figure 1**.

### Data preparation and patient selection

**Figure 2:**
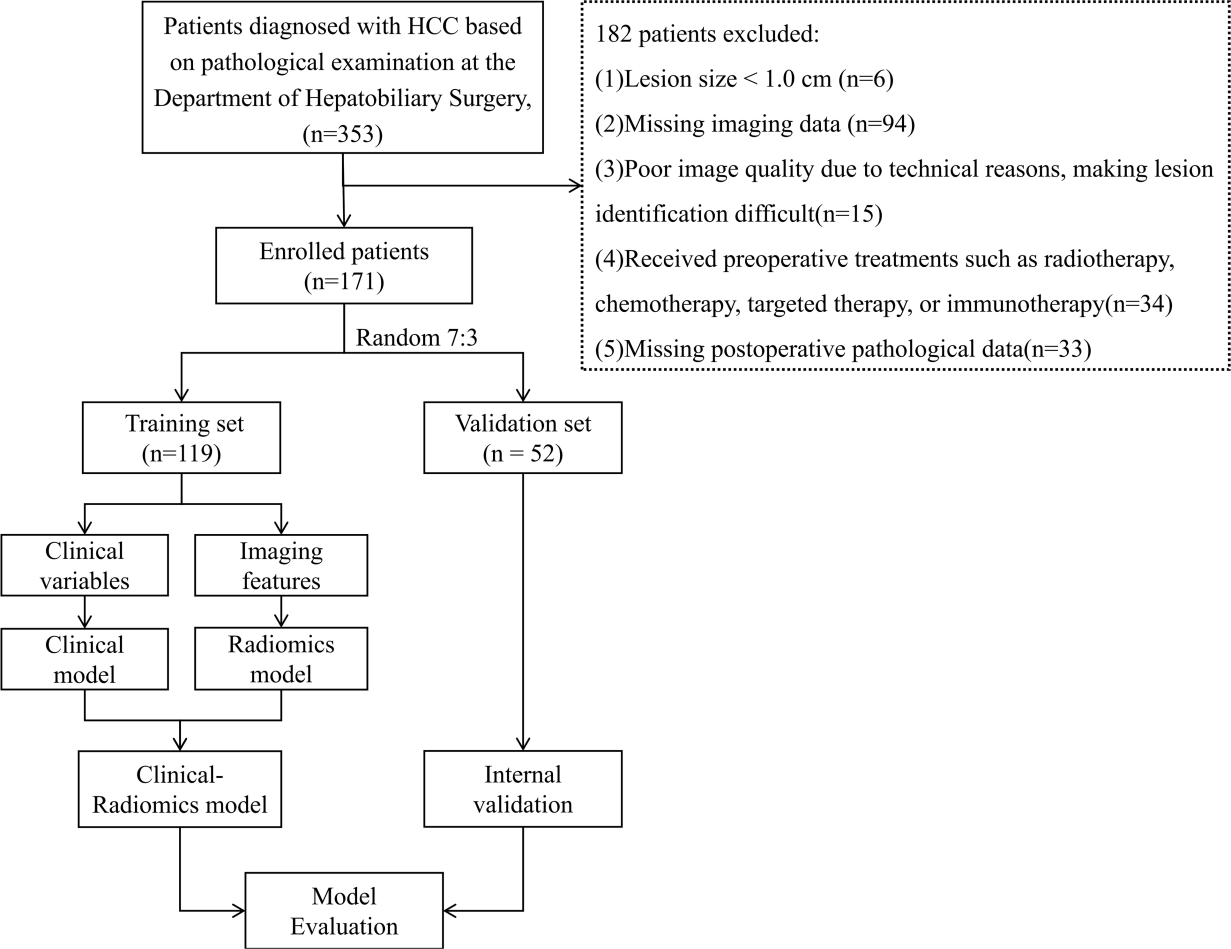
Flowchart of patient selection. HCC, hepatocellular carcinoma.

The Ethics Committee of Northern Theater General Hospital approved this retrospective study conducted at a single center (Ethics No: Y(2024)028). Between 2017 and 2024, 353 consecutive HCC patients who underwent surgical treatment at the Hepatobiliary Surgery Department of Northern Theater General Hospital were included. The inclusion criteria were: (1) a confirmed postoperative pathological diagnosis of HCC; and (2) contrast-enhanced CT of the liver performed within 1 month before surgery. The exclusion criteria were: (1) lesion size <1.0 cm; (2) missing imaging or clinical data; (3) poor image quality that hindered lesion identification; (4) history of preoperative treatments such as radiotherapy, chemotherapy, targeted therapy, or immunotherapy; and (5) missing postoperative pathological data (**Figure 2**). After applying the inclusion and exclusion criteria.

Baseline data collected included age, sex, hepatitis virus infection, Barcelona Clinic Liver Cancer staging (30), liver cirrhosis status (31), presurgical blood panels, clotting function, hepatic biochemical markers, albumin-bilirubin (ALBI) score, and serum AFP levels at admission. The calculation method for the ALBI score is provided in the **Supplementary Material**.

### Pathological diagnosis

All tissue sections were reviewed by two pathologists, each with > 5 years of experience in liver pathology. The presence of intratumoral TLSs was morphologically assessed using H&E-stained histopathological slides. Any disagreements between the two pathologists were resolved by a third senior pathologist (associate chief or higher). Intratumoral TLSs were classified into two maturity stages: lymphoid aggregates (Agg) and lymphoid follicles (FOL) (17). Our analysis strictly adhered to the established classification and diagnostic criteria for intratumoral TLSs proposed by Julien Calderaro and colleagues (23).

Aggregates: Vague, ill-defined clusters of lymphocytes.

Primary follicles (FL-I) are round, well-defined clusters of lymphocytes without a germinal center. Secondary follicles (FL-II) are follicles that contain a visible germinal center.

Tumors containing at least one intratumoral TLS were classified as TLSs-positive, while those without were classified as TLSs-negative.

### CT image acquisition, volume of interest segmentation, and extraction of radiological features

The process of CT image acquisition is detailed in the supplementary document. The images were imported into ITK-SNAP (version 3.8.0). An experienced physician specializing in abdominal imaging manually delineated the VOI for each tumor layer along the tumor boundaries using ITK-SNAP. Before model construction, imaging data from 20 randomly selected patients were used. The first physician reoutlined the VOI after 1 month. A second experienced physician specializing in abdominal imaging also delineated the VOI for the same 20 patients. Observer agreement was assessed both between different raters (inter-observer) and within the same rater (intra-observer) through ICC calculations. Features with an ICC > 0.75 were selected for further analysis. Both physicians performed their assessments blinded to clinical and pathological records. Feature extraction from medical images was conducted using Pyradiomics (version 3.0.1), an open-source computational package. From both arterial and portal venous phases, 1, 502 radiomic features were derived (**Supplementary Figure 1**). Features were labeled with an “A” (arterial) or “V” (venous) prefix and merged using a pre-fusion approach, resulting in 3, 004 radiomic features per patient.

### Radiomics feature selection, clinical-radiomics model construction, and evaluation

A 7:3 random split was used to divide HCC patients into training and validation datasets. Univariate analysis was conducted on the clinical data from the training set, and variables with P < 0.1 were included in a multivariate logistic regression to develop a clinical model. For feature dimensionality reduction, statistically insignificant features were initially excluded using t-tests (P < 0.05), followed by LASSO regression with 10-fold cross-validation. The λ value was selected based on the minimum criterion to identify the most predictive radiomic features.

After evaluating multiple machine learning-derived radiomics models, the highest-performing model was enhanced by incorporating independent prognostic factors to develop a combined model. The predictive model’s calibration was assessed through (1) the generation of calibration curves comparing predicted values with actual TLSs expression, and (2) the computation of SHAP values to explain feature contributions. The model’s goodness of fit was evaluated using the Hosmer-Lemeshow test. Clinical applicability was assessed via decision curve analysis (DCA).

### Statistical analysis

We analyzed the data using SPSS version 27.0 (IBM Corporation). A p-value < 0.05 was considered statistically significant. For qualitative data, we applied the chi-square test and Fisher’s exact test. Quantitative variables with a normal distribution were analyzed using the t-test, and results were presented as mean ± standard deviation. For data not normally distributed, we used the rank sum test and reported results as median (interquartile range). High-dimensional feature selection, model construction, and evaluation—including LASSO regression, cross-validation, and machine learning methods—along with model interpretability analysis (SHAP analysis), were performed using Python version 3.9.

## Results

### Clinical characteristics

The study included 171 HCC patients, with 119 (69.6%) in the training set and 52 (30.4%) in the validation set. The prevalence of intratumoral TLSs was balanced between the two sets, with 52.94% (63/119) TLSs-positive cases in the training set and 53.85% (28/52) in the validation set (P = 0.914 for inter-set difference).

Notably, TLSs-positive patients demonstrated significantly more favorable clinical profiles (**Table 1**).

**Table 1:** Characteristics of patients in the training and validation sets.

Variables	Training set (n=119)		*P*	Validation set (n=52)		*P*
	TLSs-negative (n=56)	TLSs-positive (n=63)		TLSs-negative (n=24)	TLSs-positive (n=28)	
Age (y), mean ± SD	58.57 ± 9.66	59.29 ± 9.51	0.686	57.38 ± 10.11	58.64 ± 9.63	0.646
WBC (×10^9^/L), M (Q_1_, Q_3_)	5.00 (3.80, 5.73)	4.60 (3.70, 6.70)	0.617	5.45 (4.77, 6.73)	5.50 (4.40, 6.65)	0.776
RBC (×10^12^/L), M (Q_1_, Q_3_)	4.47 (4.00, 4.77)	4.44 (4.08, 4.71)	0.964	4.61 (4.20, 4.81)	4.42 (4.04, 4.75)	0.287
PLT (×10^9^/L), M (Q_1_, Q_3_)	144.00 (115.50, 169.50)	153.00 (95.00, 224.00)	0.239	163.50 (146.75, 197.00)	164.00 (134.00, 216.00)	0.640
ALT (U/L), M (Q_1_, Q_3_)	34.28 (24.00, 47.18)	28.72 (22.77, 42.52)	0.270	38.56 (20.88, 44.39)	26.60 (18.14, 39.15)	0.204
AST (U/L), M (Q_1_, Q_3_)	32.00 (23.75, 45.75)	27.83 (21.02, 40.86)	0.322	24.76 (19.84, 34.82)	28.78 (22.62, 41.89)	0.162
PT (s), M (Q_1_, Q_3_)	13.90 (13.40, 14.50)	13.90 (13.40, 14.45)	0.784	13.60 (12.97, 13.90)	13.70 (13.20, 14.30)	0.398
APTT (s), M (Q_1_, Q_3_)	35.60 (34.35, 37.80)	36.60 (34.90, 39.40)	0.221	37.45 (34.88, 38.68)	35.45 (34.03, 38.28)	0.169
Sex, n (%)			0.828			0.359
Male	86 (72.27)	41 (73.21)		38 (73.08)	19 (79.17)	
Female	33 (27.73)	15 (26.79)		14 (26.92)	5 (20.83)	
HBV/HCV, n (%)			0.939			1.000
Negative	28 (23.53)	13 (23.21)		10 (19.23)	5 (20.83)	
Positive	91 (76.47)	43 (76.79)		42 (80.77)	19 (79.17)	
Liver cirrhosis, n (%)			0.500			0.111
Negative	76 (63.87)	34 (60.71)		38 (73.08)	15 (62.50)	
Positive	43 (36.13)	22 (39.29)		14 (26.92)	9 (37.50)	
BCLC, n (%)			0.435			0.882
0-A	95 (79.83)	43 (76.79)		44 (84.62)	21 (87.50)	
B	24 (20.17)	13 (23.21)		8 (15.38)	3 (12.50)	
AFP≥400ng/ml, n (%)			**0.027**			**0.013**
Negative	78 (65.55)	31 (55.36)		27 (51.92)	8 (33.33)	
Positive	41 (34.45)	25 (44.64)		25 (48.08)	16 (66.67)	
ALBI grade≥2, n (%)			**<.001**			**<.001**
Negative	54 (45.38)	11 (19.64)		25 (48.08)	5 (20.83)	
Positive	65 (54.62)	45 (80.36)		27 (51.92)	19 (79.17)	

Data are presented as number (%), median (interquartile range), or mean ± SD.

SD, standard deviation; WBC, white blood cell; RBC, red blood cell; PLT, platelets; ALT, alanine aminotransferase; AST, aspartate aminotransferase; PT, prothrombin time; APTT, activated partial thromboplastin time; HBV, hepatitis B virus; HCV, hepatitis C virus; BCLC, Barcelona Clinic Liver Cancer Staging System; AFP, alpha-fetoprotein; ALBI, albumin-bilirubin.

The bold values represents a statistically significant difference.

Lower rates of AFP ≥ 400 ng/mL (training set: P = 0.027; validation set: P = 0.035).

Reduced prevalence of ALBI grade ≥ 2 (training set: P < 0.001; validation set: P = 0.008).

Multivariate analysis, including all variables that were significant in univariate analysis (P < 0.1), confirmed that an ALBI grade ≥ 2 is an independent negative predictor of intratumoral TLSs expression (adjusted OR: 0.32, 95% CI: 0.18–0.56; P < 0.001). Detailed results are presented in **Supplementary Table 1**.

### Feature selection and predictive performance assessment in radiomics modeling

**Figure 3:**
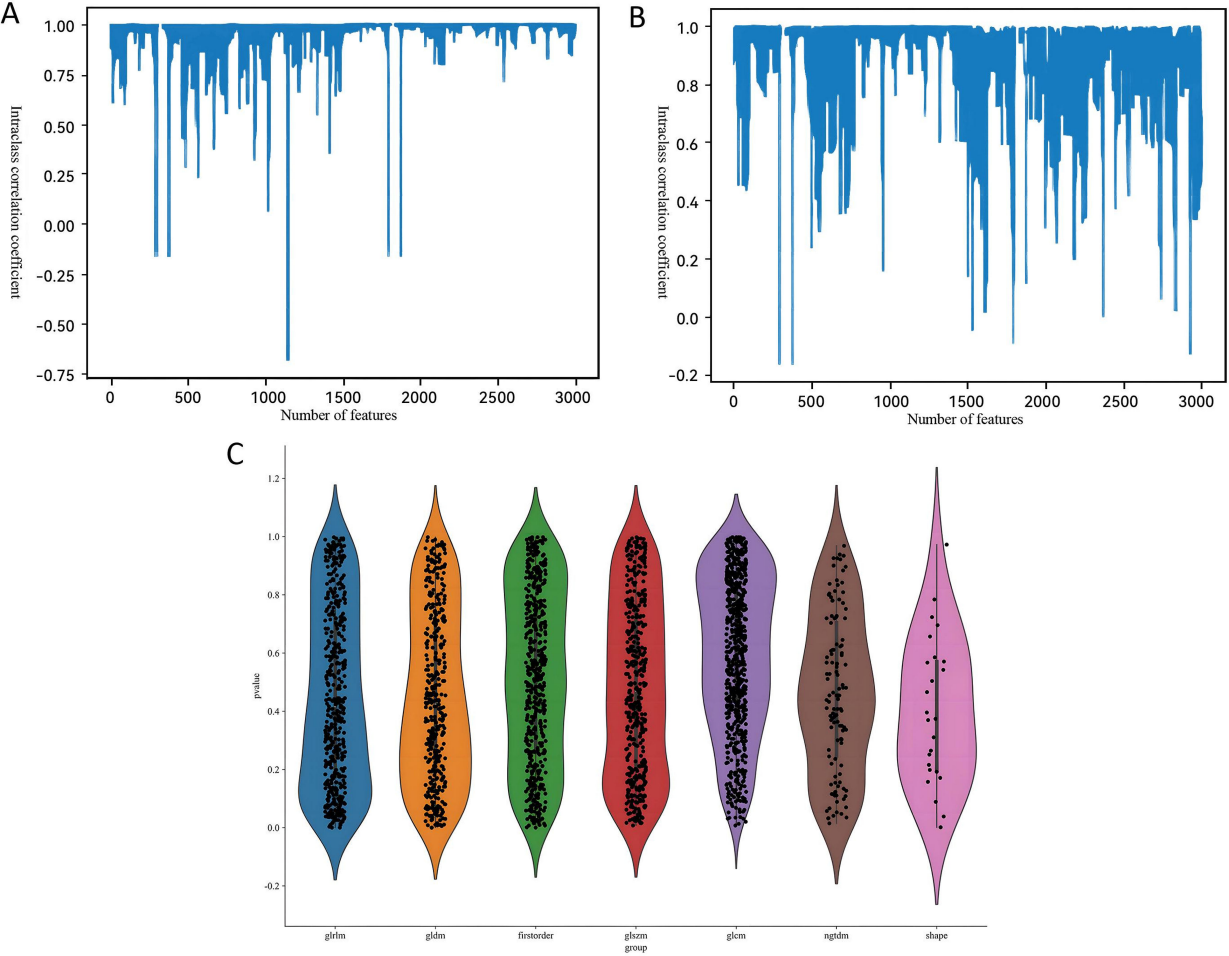
Bar charts of the intraclass correlation coefficient for **(A)** intra- and **(B)** inter-observer reliability. **(C)** Statistical plot of radiomic features.

**Figure 4:**
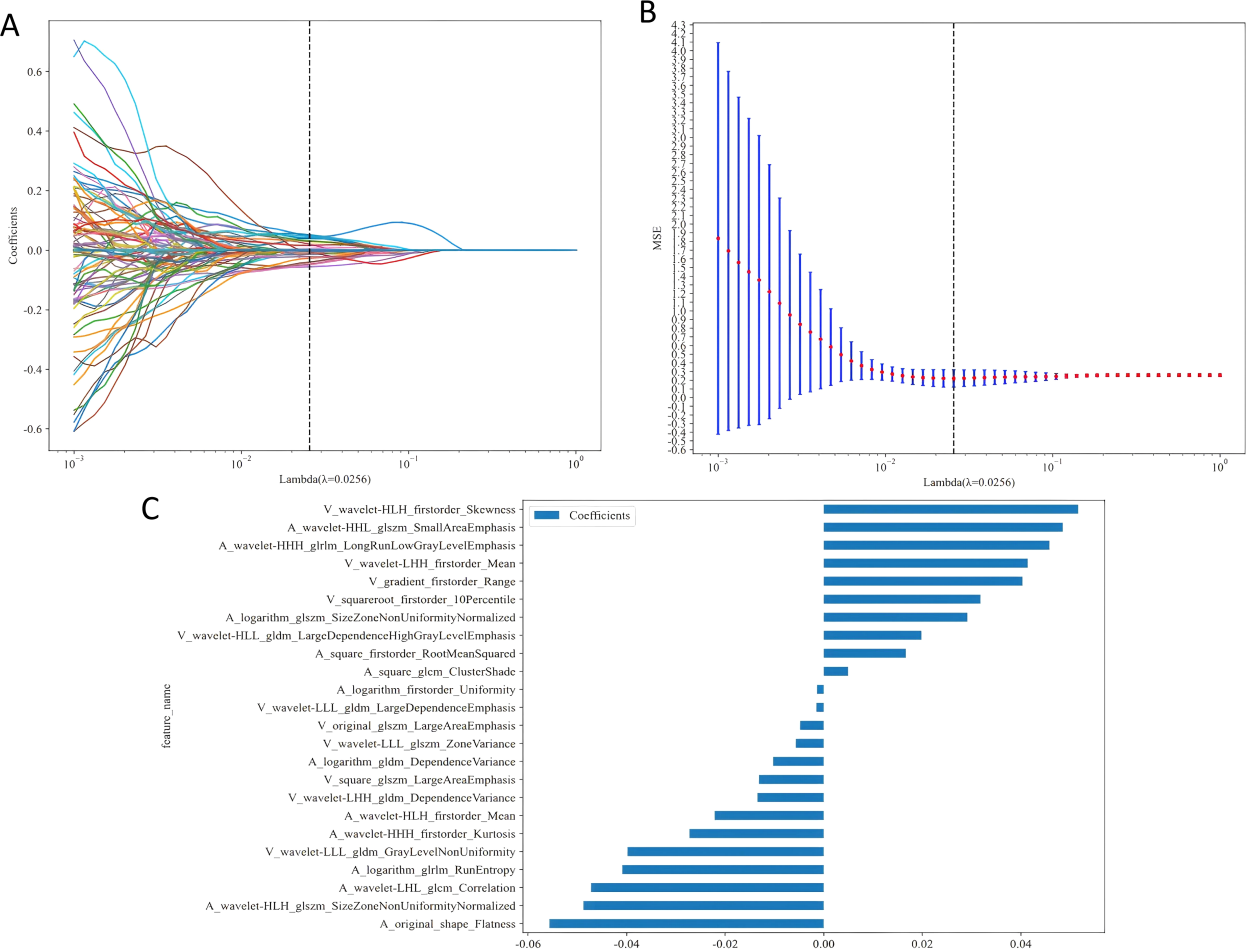
**(A)** Distribution of LASSO coefficients for each radiomic feature. **(B)** Optimal penalty coefficient (λ) for the LASSO model, determined via 10-fold cross-validation and the 1-SE rule. **(C)** Histogram of selected feature coefficients. 1-SE, One Standard Error.

Our radiomics feature selection process demonstrated high reproducibility, with 94.8% (2, 847/3, 004) and 87.4% (2, 626/3, 004) of features meeting the consistency threshold (ICC ≥ 0.75) in intra- and inter-observer analyses, respectively (**Figures 3A, B**). Ultimately, 2, 588 imaging features were retained for subsequent analysis. These features underwent rigorous statistical refinement: initial univariate screening using t-tests identified significantly discriminative features (**Figure 3C**), which were then standardized and processed via LASSO regression with 10-fold cross-validation (**Figures 4A, B**). Following final dimensionality reduction, 24 optimal predictive radiomics features were selected (13 from the arterial phase and 11 from the portal venous phase; **Figure 4C**). These features were significantly correlated with the expression of intratumoral TLSs in hepatocellular carcinoma.

### Building and utilizing clinical-radiomics prediction models

**Figure 5:**
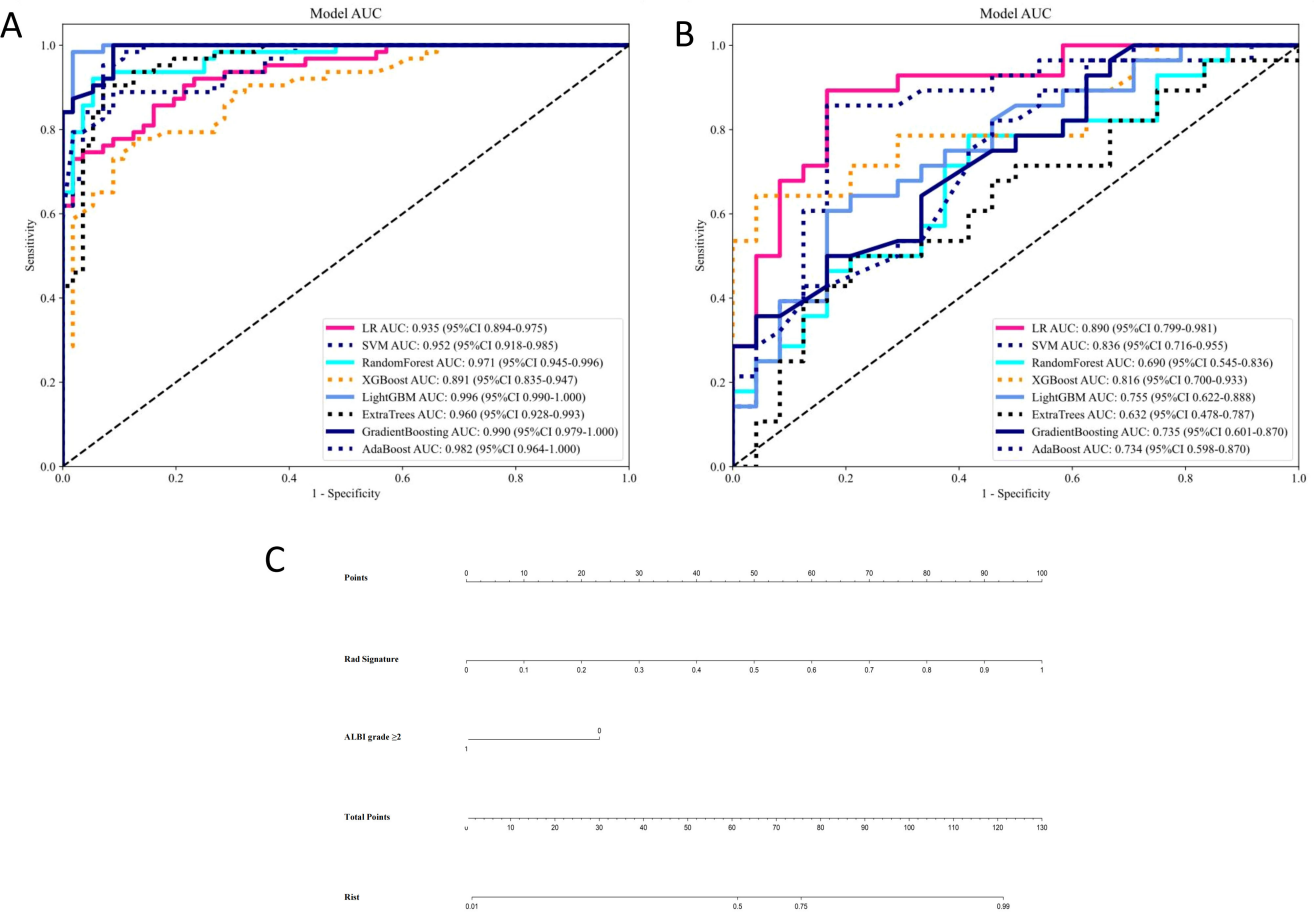
Receiver operating characteristic curves of eight machine learning-based radiomics models for predicting intratumoral tertiary lymphoid structures for the **(A)** training and **(B)** validation sets. **(C)** Nomogram combining albumin-bilirubin score ≥2 and Rad Signature. LR, logistic regression; SVM, support vector machine; XGBoost, eXtreme Gradient Boosting; LightGBM, Light Gradient Boosting Machine; AdaBoost, Adaptive Boosting.

**Figure 6:**
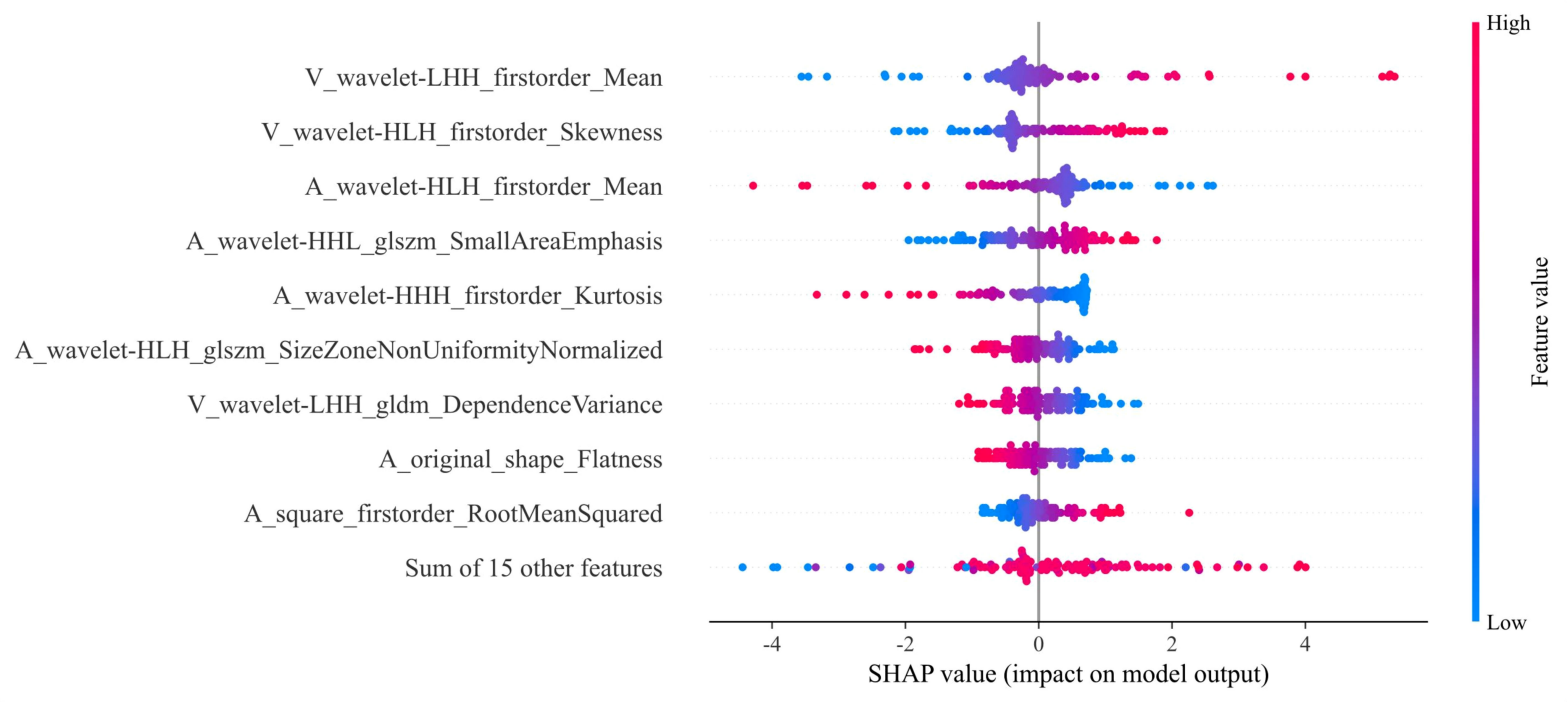
Mean SHAP values for each radiomic feature. SHAP, SHapley Additive exPlanations.

**Figure 7:**
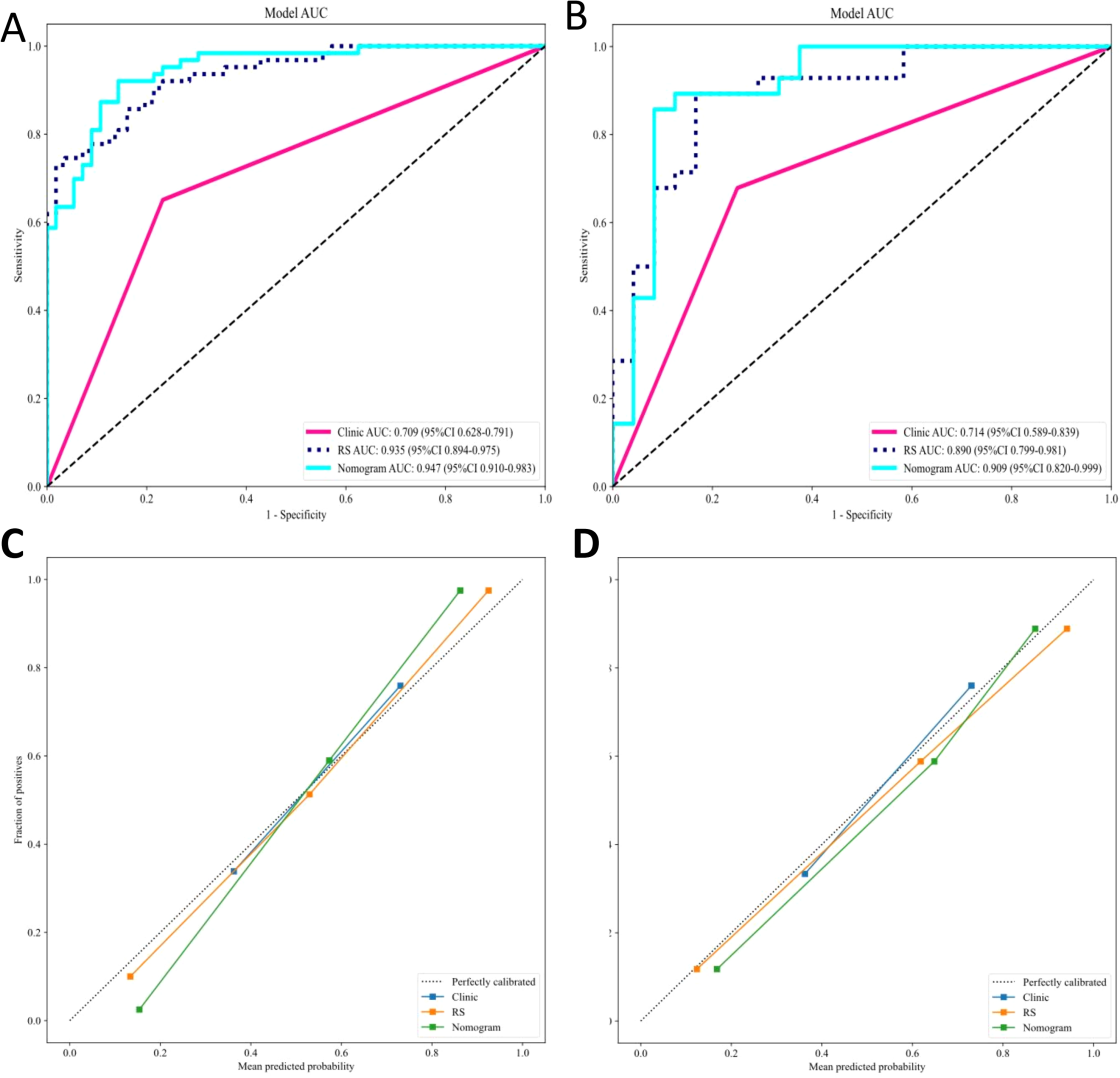
ROC and calibration curves for the clinical, radiomics, and combined models. **(A)** ROC curves for the **(A)** training and **(B)** validation sets. Calibration curves for the nomograms in the **(C)** training and **(D)** validation sets. ROC, receiver operating characteristic; RS, Rad Signature.

Our comprehensive evaluation of eight machine learning algorithms identified logistic regression as the optimal radiomics model, demonstrating superior predictive performance in both the training set (AUC 0.935, 95% CI 0.894–0.975) and the validation set (AUC 0.890, 95% CI 0.799–0.981) (**Figures 5A, B**, **Table 2**). SHAP analysis provided transparent interpretation of feature contributions (**Figure 6**), with detailed local explanations available through waterfall and force plots (**Supplementary Figures 2**). The integration of radiomic features with clinical predictors (ALBI grade ≥ 2) in our combined nomogram (**Figure 5C**) achieved outstanding discrimination, with AUCs of 0.947 (95% CI 0.910–0.983) in the training set and 0.909 (95% CI 0.820–0.999) in the validation set—representing significant improvements over the clinical-only model’s performance (training AUC 0.709, validation AUC 0.714; **Figures 7A, B**). The model’s clinical utility was further supported by excellent calibration (**Figures 7C, D**) and favorable decision curve analysis results (**Supplementary Figure 3**), confirming its reliability for predicting intratumoral TLSs expression in HCC patients.

**Table 2:** Diagnostic performance of eight machine learning models in the training and validation sets.

Set	Model	Accuracy	Sensitivity	Specificity	AUC (95% CI)
Training	LR	0.84	0.71	0.98	0.93 (0.78–0.98)
	SVM	0.89	0.83	0.96	0.95 (0.92–0.98)
	RandomForest	0.92	0.90	0.95	0.97 (0.95–1.00)
	XGBoost	0.82	0.76	0.87	0.89 (0.83–0.95)
	LightGBM	0.97	0.97	0.98	1.00 (0.99–1.00)
	ExtraTrees	0.90	0.87	0.93	0.96 (0.92–0.99)
	GradientBoosting	0.93	0.95	0.91	0.99 (0.97–1.00)
	AdaBoost	0.92	0.92	0.93	0.98 (0.96–1.00)
Validation	LR	0.85	0.86	0.83	0.89 (0.80–0.98)
	SVM	0.83	0.82	0.83	0.84 (0.72–0.95)
	RandomForest	0.67	0.75	0.58	0.69 (0.55–0.84)
	XGBoost	0.77	0.61	0.96	0.82 (0.70–0.93)
	LightGBM	0.69	0.57	0.83	0.76 (0.62–0.89)
	ExtraTrees	0.62	0.46	0.79	0.63 (0.48–0.79)
	GradientBoosting	0.63	0.46	0.83	0.74 (0.60–0.87)
	AdaBoost	0.67	0.79	0.54	0.73 (0.60–0.87)

AUC, area under the curve; ACC; LR, logistic regression; SVM, support vector machine; XGBoost, eXtreme Gradient Boosting; LightGBM, Light Gradient Boosting Machine; AdaBoost,Adaptive Boosting.

## Discussion

Surgical resection, liver transplantation, and transarterial chemoembolization (TACE) remain cornerstone treatments for HCC; however, their efficacy is often limited in advanced-stage disease (32). Accumulating evidence highlights intratumoral immune cell infiltration as a key predictor of immunotherapy response (33), with intratumoral TLSs playing a particularly critical role. The density of TLSs correlates with improved pathological responses and prolonged recurrence-free survival. Notably, the presence of atypical TLSs in tumor regression zones may promote T-cell memory formation, further underscoring their importance in HCC immunotherapy (34).

The formation of TLS alters the spatial architecture within liver cancer tumors. For instance, texture features may reflect the microscopic complexity resulting from lymphocyte aggregation, while morphological features may correspond to the overall macroscopic structure of TLS. Although these microscopic changes are not directly visible to the naked eye in imaging, they can be decoded using quantitative radiomics algorithms. Therefore, this study employs machine learning to investigate the correlation between radiomics features of HCC and the pathological characteristics of TLS. By integrating radiomics features with clinical data, we aim to conduct an in-depth exploration of the intrinsic relationship between intratumoral TLS and HCC progression.

Our research found that the occurrence rate of intratumora TLSs among tumor cells in HCC (53.22%, 91/171) was consistent with previously reported rates (29, 35). Multivariate analysis indicated that the ALBI score was the only clinical parameter independently associated with the presence of TLSs within the tumor. The study confirmed that the ALBI score has an independent prognostic role in HCC. A higher ALBI score (2 or 3 points, compared to 1 point) independently indicates a threefold increase in the risk of death (36). Moreover, a higher ALBI score is associated with an increased risk of postoperative recurrence (37), which may reflect impaired immune surveillance in decompensated liver disease. Our data show a significant negative correlation between the ALBI score and the density of TLSs within the tumor (p < 0.05). Better liver function (lower ALBI score) may imply healthier immune system function, supporting a more effective anti-tumor immune response and the formation of TLSs. Conversely, liver dysfunction may hinder the generation and maintenance of TLSs through systemic inflammation or immunosuppressive states. Patients with ALBI grade 1 have better immune function, promoting the generation of intratumoral TLSs and a better prognosis.

ML has emerged as a transformative tool in the medical field, demonstrating remarkable potential in various omics domains of liver cancer (26). Its application in liver cancer diagnosis is particularly prominent. Gao et al. developed an ML model that utilizes preoperative contrast-enhanced CT imaging and clinical data to distinguish malignant liver tumors. The model achieved an accuracy rate of 86.2% and an AUC value of 0.893, enabling the differentiation between HCC and intrahepatic cholangiocarcinoma (ICC) (38).

ML also exhibits outstanding practicality in predicting key histopathological features of liver cancer. MVI, an important predictor of postoperative recurrence, has long been a focus of radiomics research. Multiple ML models incorporating radiomics features have demonstrated excellent performance in predicting MVI (39, 40). These advancements highlight the ability of machine learning to extract clinically relevant information from routine imaging data, indicating that ML can obtain certain pathological features of tumors from imaging materials.

To date, no studies have developed ML models combining CT radiomics and clinical data to predict intratumoral TLSs expression in HCC. However, pioneering work by Xu et al. demonstrated ML’s capability to predict TLSs in intrahepatic cholangiocarcinoma (ICC) using preoperative contrast-enhanced CT portal phase and multiphase MRI images (41). Subsequent research expanded on this approach, employing multiphase MRI and clinical data from 192 patients to predict intratumoral TLSs in ICC (42). For HCC specifically, prior investigations have utilized contrast-enhanced CT semantic features combined with clinical data to predict intratumoral TLSs patterns, achieving an average AUC of 0.75 through five-fold cross-validation (43). These foundational studies not only validate our methodological approach but also confirm the feasibility of preoperative TLSs prediction. However, a recent study focused on the density of the peritumoral TLS (pTLS) around the tumors of HCC patients and their role in predicting prognosis and immune treatment response. By integrating multi-omics data (including spatial transcriptomics and RNA sequencing) and multi-center imaging data, the study identified key regulatory factors (CXCL9/10) associated with high pTLS density and developed a non-invasive classifier based on MRI imaging biomarkers to accurately predict the density of pTLS (44).

Our study represents the first successful development of machine learning models that integrate contrast-enhanced CT radiomics with clinical parameters to predict intratumoral TLSs expression in HCC. While our clinical-only model achieved comparable performance (AUC ≈ 0.75) to previous HCC studies, the radiomics model and combined model demonstrated significantly superior predictive capability, with the integrated nomogram (combining ALBI score and radiomic features) emerging as the optimal tool for preope.

This study has several limitations that should be considered. First, the retrospective design may introduce selection bias, and since our inclusion criteria were restricted to surgical patients, the findings are currently only applicable to resectable HCC cases - additional studies are needed to evaluate generalizability to advanced/unresectable HCC populations. Second, as a single-center retrospective analysis, external validation through prospective multicenter trials will be essential to confirm our results and support clinical translation. Third, while our radiomics model demonstrated promising results using contrast-enhanced CT alone, incorporating preoperative MRI in future studies could further improve predictive performance and provide stronger evidence for clinical implementation through multimodal imaging integration. Finally, due to the retrospective nature of this study, we were unable to prepare traditional clear pathological section images. Although we have made every effort to provide detailed textual descriptions, this deficiency still exists. In future related studies, attention should be paid to the collection and presentation of pathological images. In the future, we will continue to increase the number of patients. We will also analyze the relationship between the presence of TLSs and the prognosis of HCC patients and verify its correlation with the efficacy of immunotherapy. At the same time, we may explore the connections between imaging features such as MRI and PET-CT and TLSs in HCC patients. The integrated model constructed in this study aims to become a highly accurate, non-invasive “virtual biopsy” tool, which not only can guide the clinical decision-making of immunotherapy for patients with hepatocellular carcinoma but also will promote the exploration of the underlying mechanisms of the tumor immune microenvironment.

## Conclusion

In summary, the developed machine learning models for preoperative prediction of intratumoral TLSs expression in HCC by integrating radiomic features extracted from arterial and portal phase contrast-enhanced CT images with relevant clinical data. Our models - including clinical-only, radiomics-only, and combined approaches - consistently demonstrated robust predictive performance across both training and validation sets. These findings suggest that our ML framework could serve as a valuable clinical tool for noninvasive intratumoral TLSs assessment prior to surgery, potentially guiding personalized treatment selection and optimizing therapeutic decision-making for HCC patients. The strong performance across all model types highlights the complementary value of both imaging biomarkers and clinical parameters in predicting this important immunological feature of HCC.

## Data Availability

The raw data supporting the conclusions of this article will be made available by the authors, without undue reservation.
